# Late-life depressive symptoms and white matter structural integrity within older Black adults

**DOI:** 10.3389/fnagi.2023.1138568

**Published:** 2023-05-02

**Authors:** Debra A. Fleischman, Konstantinos Arfanakis, Sue E. Leurgans, Shengwei Zhang, Melissa Lamar, S. Duke Han, Victoria N. Poole, Namhee Kim, David A. Bennett, Lisa L. Barnes

**Affiliations:** ^1^Rush Alzheimer’s Disease Center, Chicago, IL, United States; ^2^Department of Neurological Sciences, Rush University Medical Center, Chicago, IL, United States; ^3^Department of Psychiatry and Behavioral Sciences, Rush University Medical Center, Chicago, IL, United States; ^4^Department of Diagnostic Radiology and Nuclear Medicine, Rush University Medical Center, Chicago, IL, United States; ^5^Department of Biomedical Engineering, Illinois Institute of Technology, Chicago, IL, United States; ^6^Department of Preventive Medicine, Rush University Medical Center, Chicago IL, United States; ^7^Department of Family Medicine and Neurology, Keck School of Medicine, Los Angeles, CA, United States; ^8^Department of Psychology, University of Southern California, Los Angeles, CA, United States; ^9^School of Gerontology, University of Southern California, Los Angeles, CA, United States; ^10^Department of Orthopedic Surgery, Rush University Medical Center, Chicago, IL, United States

**Keywords:** Black, African-American, late-life depressive symptoms, brain structure, diffusion-tensor imaging

## Abstract

**Introduction:**

Older Black adults experience a high burden of depressive symptoms and cerebrovascular disease but the specific neurobiological substrates underlying the association between late-life depressive symptoms and brain integrity are understudied, particularly in within-group designs.

**Methods:**

Using the Center for Epidemiologic Studies Depression Scale and diffusion-tensor imaging, within-Black variation in the association between late-life depressive symptoms and white matter structural integrity was examined in 297 older Black participants without dementia that were enrolled across three epidemiological studies of aging and dementia. Linear regression models were used to test associations with DTI metrics (fractional anisotropy, trace of the diffusion tensor) as the outcomes and depressive symptoms as the predictor, while adjusting for age, sex, education, scanner, serotonin-reuptake inhibitor use, total volume of white-matter hyperintensities normalized by intracranial volume, and presence of white-matter hyperintensities at the voxel level.

**Results:**

Higher level of self-reported late-life depressive symptoms was associated with greater diffusion-tensor trace (reduced white matter integrity) in connections between commissural pathways and contralateral prefrontal regions (superior and middle frontal/dorsolateral prefrontal cortex), association pathways connecting dorsolateral prefrontal cortex with insular, striatal and thalamic regions, and association pathways connecting the parietal, temporal and occipital lobes and the thalamus.

**Discussion:**

This study demonstrated a discernable pattern of compromised white matter structural integrity underlying late-life depressive symptoms within older Black adults.

## Introduction

Depressive symptoms occurring in late life are associated with several adverse health outcomes including cerebrovascular disease and stroke (Pan et al., 2011; Taylor et al., 2013; Aizenstein et al., 2016; Van Agtmaal et al., 2017), cognitive impairment and dementia (Panza et al., 2010; Diniz et al., 2013; Barnes, 2014; Rashidi-Ranjbar et al., 2020), functional disability (Callahan et al., 1998; Wassink-Vossen et al., 2019), morbidity (Lamar et al., 2015; Cummings et al., 2016; Cho and Hamler, 2021), and all-cause mortality (Saz and Dewey, 2001; Wei et al., 2019). Depressive symptoms in older adults have been linked with brain integrity in a number of studies (reviewed in Alexopoulos, 2019) but more work is needed to identify specific neurobiological substrates underlying this association, and this is particularly true for older Black adults who have a high prevalence of late-life depressive symptoms (Skarupski et al., 2005; Pickett et al., 2013) but are understudied generally and in the neurologic (Robbins et al., 2022), neuroimaging (Carmichael and Newton, 2019) and psychiatric (Price et al., 2022) literatures.

Older Black adults experience sociocultural and environmental exposures linked to depression, such as discrimination, poverty, and neighborhood disadvantage (Barnes et al., 2012a; Mezuk et al., 2013; Hastings and Snowden, 2019; Scheuermann et al., 2020), and are disproportionately burdened by depressive symptoms (Skarupski et al., 2005; Walsemann et al., 2009; Liang et al., 2011; Pickett et al., 2013; Reinlieb et al., 2014; but see Erving et al., 2019) and their associated adverse health outcomes (Prabhakaran et al., 2008; Turner et al., 2015; Steenland et al., 2016; Kalyani et al., 2017; Rajan et al., 2019; Deere and Ferdinand, 2020; Cho and Hamler, 2021; Virani et al., 2021). Older Black adults also have a higher brain white-matter hyperintensity burden (Brickman et al., 2008; Morris et al., 2009), a measure thought to generally reflect small vessel-related injury (Schmahmann et al., 2008; Wardlaw et al., 2015), yet the many MRI studies linking late-life depressive symptoms to brain integrity have mostly employed all-White or mixed samples. Focusing on an all-Black sample allows a deeper understanding of within-Black variation in the association between late-life depressive symptoms and brain integrity that could potentially inform the development of programs that reduce racial disparities in affective health (Mezuk et al., 2013; Pickett et al., 2013; Price et al., 2022) and lead to race-relevant targeted treatments.

One candidate neurobiological substrate underlying late-life depressive symptoms is altered brain white matter structural integrity. Reduced white matter structural integrity is commonly measured on *in vivo* MRI in older adults as visible regions of white matter hyperintensities (WMHs) on T2-weighted or FLAIR images and/or by lower diffusion anisotropy (fractional anisotropy; FA) or higher overall diffusivity (trace) of water molecules on diffusion tensor imaging (DTI). Lower diffusion anisotropy or higher diffusivity may reflect axonal injury or degeneration, vasogenic or cytotoxic edema, myelin disruption, inflammation, or other disturbances in diverse cellular mechanisms (Parekh et al., 2015; Bennett D. A. et al., 2018). These reductions in white matter structural integrity can lead to a “connectopathy” (Filley and Fields, 2016) or an interference of communication across a widely distributed neural circuitry that supports behavior (Schmahmann et al., 2008). Indeed, the link between late-life depressive symptoms and altered brain white matter structural integrity across numerous regions has been well-established in studies using all-White or mixed samples (Lamar et al., 2010; Wen et al., 2014; Smagula and Aizenstein, 2016; Van Agtmaal et al., 2017; Alexopoulos, 2019; Kim and Han, 2021), but to date, this association has not been examined within a large, solely Black, epidemiologic sample. In this study, self-report of late-life depressive symptoms was measured using the Center for Epidemiologic Studies Depression (CES-D) scale (Kohout et al., 1993) and white matter structure was interrogated voxel-wise with DTI, in 297 older Black participants without dementia, from three large epidemiological studies of aging and dementia.

## Materials and methods

### Participants

Participants were recruited from three parent studies of aging and dementia conducted in the United States: Minority Aging Research Study (MARS; Barnes et al., 2012b), Rush Alzheimer’s Disease Center African American Clinical Core (RADC AA Core; Barnes et al., 2012b), and the Rush Memory and Aging Project (MAP; Bennett D. A. et al., 2018). MARS and RADC AA Core both consist of older Black adults recruited from churches, senior buildings and social clubs and organizations that cater to minority populations. The Rush Memory and Aging Project is a cohort of older diverse lay persons (predominantly White) recruited from retirement communities and subsidized housing facilities across the Chicago metropolitan area. Only Black adults enrolled in MAP were eligible for this analysis. All three studies recruit participants without known dementia at baseline and annual evaluations are harmonized across all studies and include a medical history, neuropsychological testing and neurological evaluation (as previously described; Barnes et al., 2012b; Bennett I. J. et al., 2018). Participants supplied all medications prescribed by a doctor, vitamins, supplements, and over-the-counter remedies and medicines taken in the 2 weeks prior to the annual evaluation. Direct visual inspection of all containers of prescription and over-the-counter agents allowed for medication documentation. Medications were then coded using the Medi-Span Drug Data Base system (Medi-Span Inc., 1995). Three-tesla (3 T) neuroimaging was added to the parent studies between 2009 and 2012. All studies followed the ethical standards set in the 1964 Declaration of Helsinki and its later amendments and were approved by the Institutional Review Board of Rush University Medical Center.

**Figure 1:**
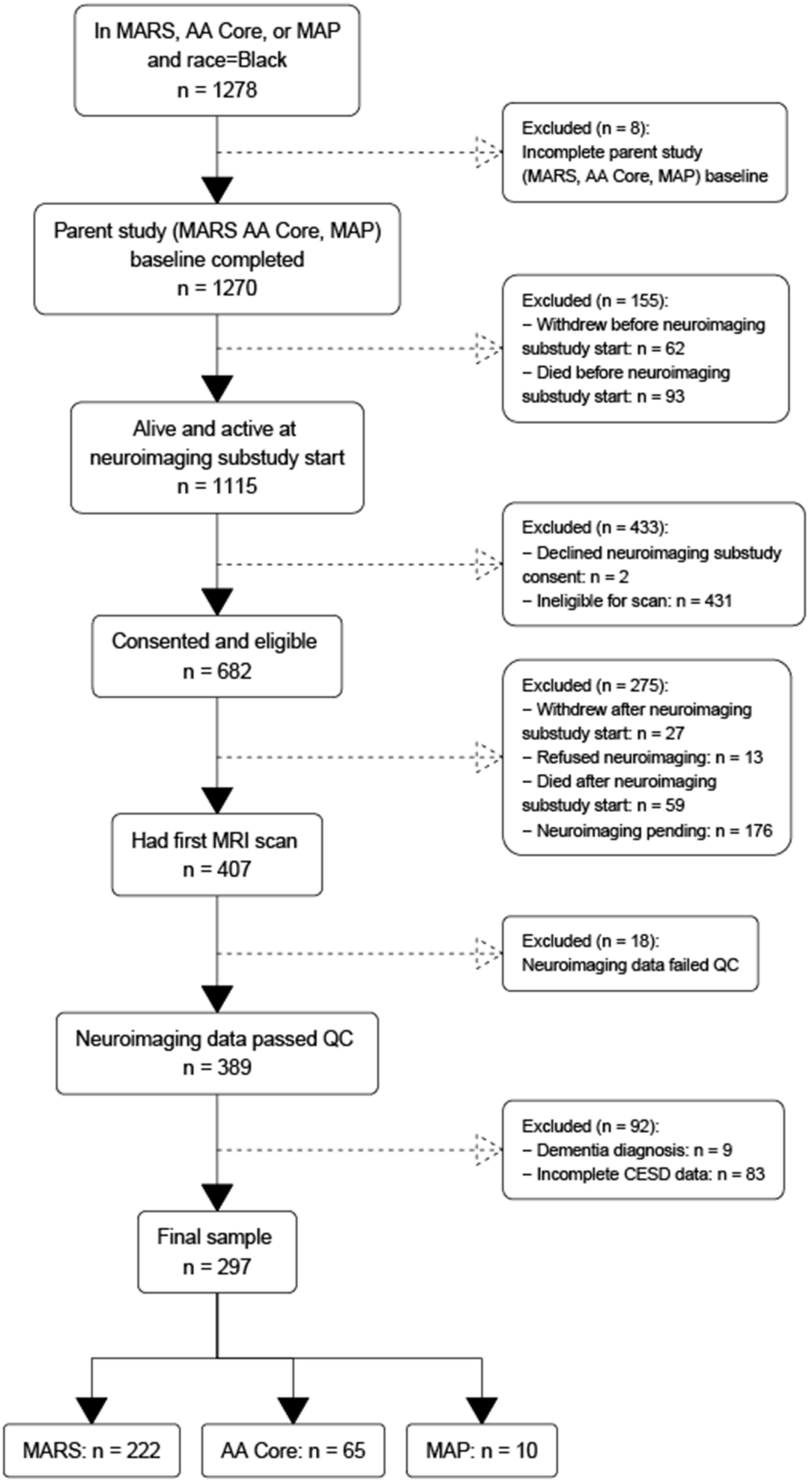
Flowchart of participant recruitment across three parent epidemiological studies of aging and dementia conducted in the United States.

Eligible participants self-identified as Black, successfully completed at least one MRI acquisition that passed quality-control (QC), completed CES-D assessments, and had no history of dementia at or before the first MRI session. To identify these participants, we began with all participants in the three parent studies described above (Figure 1).

This yielded a group of 1,270 participants that had completed baseline evaluation at the time of analysis (analytic baseline). Of these 1,270 participants, 62 withdrew and 93 died before analytic baseline, leaving a sample of 1,115 participants. Of these 1,115, 431 were ineligible to participate for various reasons (e.g., MRI contraindications such as metal in the body and claustrophobia, living out of the geographic area, physical limitations such as frailty or cognitive limitations such as dementia) and another two declined to consent, leaving 682 that were consented. Of these 682 participants, another 40 withdrew or refused, 59 died before they had their first scan, and 176 had their first scan pending, leaving 407 participants that received their initial MRI scan. Of these 407, 18 scans failed quality control resulting in 389 participants with MRI data. Of these 389 participants, nine were excluded with a diagnosis of dementia based on standard clinical evaluation (Barnes et al., 2012b; Bennett D. A. et al., 2018) and NINCDS/ADRDA diagnostic criteria (McKhann et al., 1984), and 83 were excluded for missing CES-D data. The final sample comprised 297 participants: 222 from MARS, 65 from RADC AA Core, and 10 from MAP. To examine for selection bias, we compared the analytic sample to (1) Black participants across all cohorts regardless of neuroimaging status, (2) Black participants that left the parent study before neuroimaging was begun, and (3) Black participants that were eligible for neuroimaging but were not scanned. The analytic sample had milder depressive symptoms, higher MMSE scores (within the normal range of 24 and above) and less SSRI medication use.

### Assessment of depressive symptoms

The 10-item version of the Center for Epidemiologic Studies Depression (CES-D; Kohout et al., 1993) scale was used to measure late-life depressive symptoms. This version was derived from the original 20-item version (Radloff, 1977), which has been shown to have measurement equivalency in Black adults (Nguyen et al., 2004). The 10-item version has similar factor structure as the 20-item version and acceptable reliability (Kohout et al., 1993).

### Image acquisition

MRI data were collected on a 3 T Philips MRI scanner and a 3 T Siemens scanner using a 3D magnetization prepared rapid acquisition gradient echo (MPRAGE) sequence, a 2D T2-weighted fluid-attenuated inversion recovery (FLAIR) sequence, and a 2D spin-echo echo-planar diffusion-weighted sequence. The parameters on the 3 T Philips scanner were: for MPRAGE TR = 8 ms, TE = 3.7 ms, TI = 955 ms, flip-angle = 8°, field of view = 240 mm × 228 mm, 181 sagittal slices, acquired voxel size = 1 × 1 × 1 mm^3^, and an acceleration factor of 2; for FLAIR TR = 9 s, TE = 90 ms, TI = 2,500 ms, field of view = 220 mm × 220 mm, 35 axial slices, acquired voxel size = 0.9 × 1.1 × 4 mm^3^, and an acceleration factor of 1.6; for DTI TR = 10.7 s, TE = 55 ms, field of view = 224 mm × 224 mm, 65 axial slices, acquired voxel size = 2 × 2 × 2 mm^3^, b = 1,000 s/mm^2^ for 40 diffusion directions and 6 b = 0 s/mm^2^ volumes. The parameters on the 3 T Siemens scanner were: for MPRAGE TR = 2.3 s, TE = 2.98 ms, TI = 900 ms, flip-angle = 9°, field of view = 256 mm × 256 mm, 176 sagittal slices, acquired voxel size = 1 × 1 × 1 mm^3^, and an acceleration factor of 2; for FLAIR TR = 9 s, TE = 150 ms, TI = 2,490 ms, field of view = 220 mm × 220 mm, 35 axial slices, acquired voxel size = 0.9 × 0.9 × 4 mm^3^, and an acceleration factor of 2; for DTI TR = 8.1 s, TE = 85 ms, field of view = 224 mm × 224 mm, 65 axial slices, acquired voxel size = 2 × 2 × 2 mm^3^, b = 1,000 s/mm^2^ for 40 diffusion directions and 6 b = 0 s/mm^2^ volumes.

### Image processing

Fractional anisotropy and the trace of the diffusion tensor, two of the most commonly used measures derived from the diffusion tensor (Alexander et al., 2007), were used to characterize white matter structural integrity. Correction of distortions in the diffusion-weighted volumes caused by eddy currents and magnetic field non-uniformities, bulk-motion correction, B-matrix reorientation, tensor fitting, and generation of FA and trace maps were accomplished with TORTOISE (http://www.tortoisedti.org; Basser and Pierpaoli, 1996; LeBihan et al., 2001; Pierpaoli et al., 2020). White matter lesions appearing hyperintense in T2-weighted images (white matter hyperintensities; WMHs) were segmented for each participant based on both MPRAGE and FLAIR data (FSL, FMRIB, University of Oxford, United Kingdom) using BIANCA (Griffanti et al., 2016), and a mask (0 and 1 s) was generated (voxels with WMH were given values of 1). The WMH mask of each participant was transformed to the space of the corresponding processed DTI data based on the transformation of the FLAIR image volume to the b = 0 s/mm^2^ volume.

### Statistical approach

#### CES-D analyses

Cumulative CES-D scores were averaged over the period beginning at parent study baseline up to and including MRI analytic baseline. Higher cumulative CES-D scores reflected more depressive symptoms. Persons with a history of any reported SSRI use over this period were identified.

#### DTI analyses

**Figure 2:**
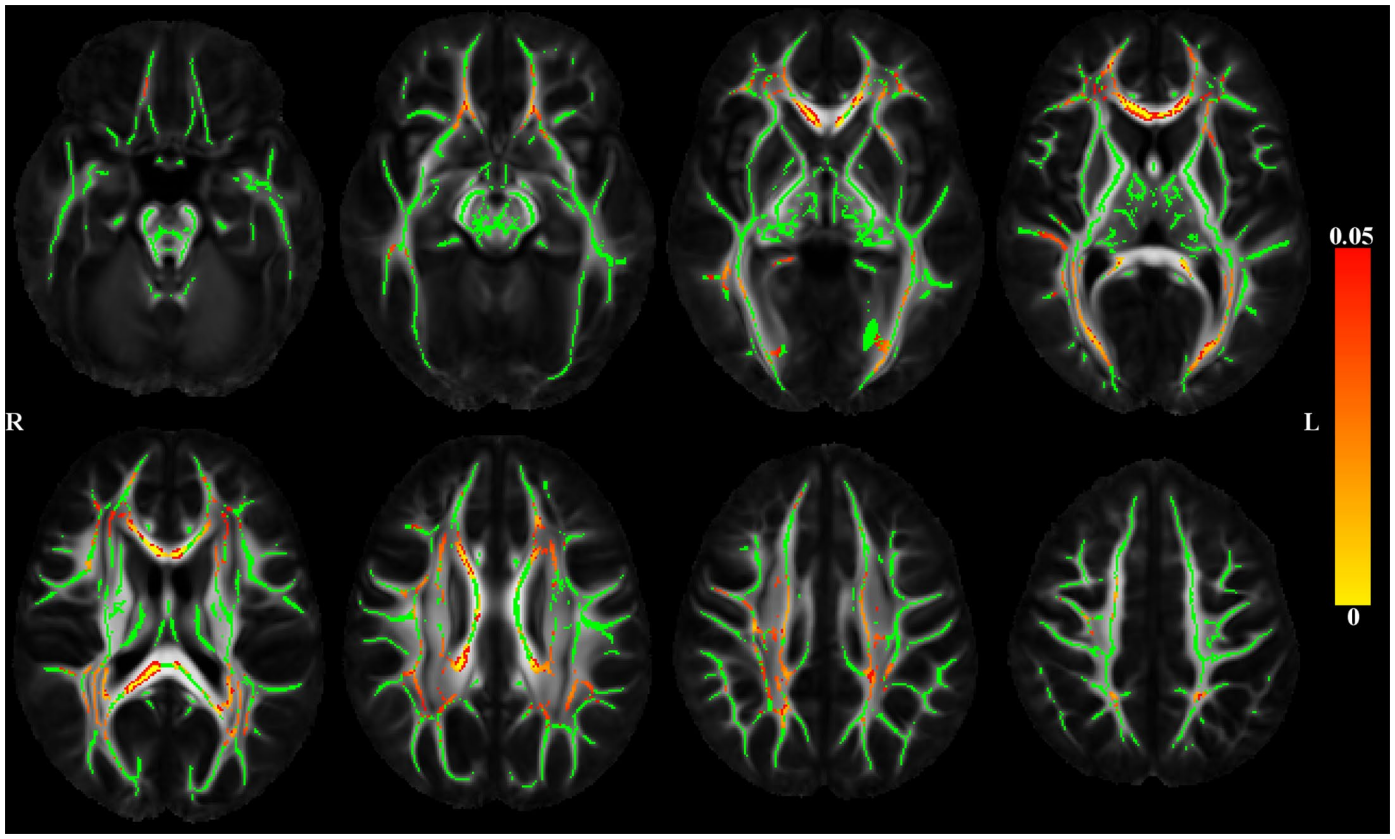
Maps of *p* values (warm color scale) showing voxels of the white matter skeleton (green color) with significant positive associations (*p* < 0.05) between depressive symptoms and the trace of the diffusion tensor, in linear regression models adjusted for age, education, sex, scanner, total volume of WMHs normalized by intracranial volume and the presence of WMH at the voxel level.

The association of DTI-derived metrics (FA, trace) with depressive symptoms was analyzed voxel-wise along the white matter skeleton (Smith et al., 2006). DTI data from individual participants were non-linearly transformed to the space of the IIT Human Brain Atlas (v.5.0; www.nitrc.org/projects/iit; Zhang and Arfanakis, 2018) using DR-TAMAS. The resulting spatial transformations were then applied to the corresponding FA maps, and local FA maxima were projected onto the IIT Human Brain Atlas (v.5.0) white matter skeleton using TBSS (Smith et al., 2006). The same projection parameters were used to project the trace and WMH mask values from the same voxels as the local FA maxima. Linear regression was then used to test the association of FA along the white matter skeleton (outcome) with depressive symptoms (predictor), while adjusting for age, sex, education, scanner, the total volume of WMHs normalized by the intracranial volume, and the presence of WMHs at the voxel level. SSRI use was added as an additional covariate in a second model. Models were then rerun using the trace of the diffusion tensor along the white matter skeleton (outcome) with depressive symptoms (predictor). The analysis was conducted using FSL Permutation Analysis of Linear Models (PALM; Winkler et al., 2015), assuming different variances across scanners and using two exchangeability blocks (one per scanner; i.e. permutations occurred only between participants imaged on the same scanner). Statistical inference was based on 1,000 permutations of the data, and tail approximation was used to accelerate the analysis (Winkler et al., 2016). Associations were considered significant at *p* < 0.05, with Family Wise Error Rate (FWER) correction. The Threshold-Free Cluster Enhancement (TFCE; Smith and Nichols, 2009) method was used to define clusters of significance. The regionconnect software^1^ was used to determine the most probable connections passing through clusters showing significant effects, according to the connectivity information contained in the IIT Human Brain Atlas (v.5.0; developed using high angular resolution diffusion imaging probabilistic tractography; Figure 2).

## Results

### Sample characteristics

Sample characteristics are shown in Table 1. Of the 297 persons in the analytic sample, 82% were female, had a mean age at the time of scan of 76 years (SD = 6 years) and a mean education of 15 years (SD = 3 years). Global cognition was within normal range (MMSE = 28/30, SD = 1.8). Participants reported an average of 1.7 depressive symptoms (SD = 2.0). Approximately 4% of the sample had a history of SSRI use.

**Table 1:** Sample characteristics (*N* = 297).

Measure	Mean	SD
Age (years)	76.3	6.2
Education (years)	15.5	3.3
# Female (%)	242/297 (81%)	
CES-D	1.73	2.04
SSRI ever use	13/297 (4.4%)	
MMSE	28	1.8

CES-D, center for epidemiologic studies depression scale; SSRI, serotonin reuptake inhibitor; MMSE, mini-mental state cognitive screening examination.

### Voxel-based DTI and late-life depressive symptoms

Figure 2 shows significant positive associations between late-life depressive symptoms and the trace of the diffusion tensor, adjusted for demographics and WMHs. Higher level of reported late-life depressive symptoms was associated with greater trace (higher diffusivity) throughout the brain. Adding SSRI use as a covariate did not change the results (data not shown).

Regionconnect generated lists of the most probable white matter connections traversing the clusters shown in Figure 2 that had significant associations between depressive symptoms and trace of the diffusion tensor. Clusters 1 and 2 were dominated by association pathways connecting the superior frontal and rostral middle frontal cortices (two regions encompassing the dorsolateral prefrontal cortex, DLPFC) with each other and with the insula, as well as with subcortical regions including the caudate, putamen, thalamus. Clusters 3 and 4 were dominated by association pathways connecting the parietal, temporal and occipital lobes and the thalamus. Cluster 5 was dominated by commissural pathways connecting mainly the superior frontal, rostral middle frontal, and superior parietal lobes across hemispheres (Table 2).

No associations were found between late-life depressive symptoms and white matter fractional anisotropy.

**Table 2:** List of most probable connections passing through the white matter clusters showing significant associations of depressive symptoms and trace of the diffusion tensor adjusting for age, education, sex, scanner, total volume of WMHs normalized by intracranial volume, and the presence of WMH at the voxel level as seen in Figure 2.

Cluster #	Connection	%
1	R thalamus and R superior frontal	3.4
	L superior frontal and R superior frontal	3.2
	R putamen and R superior frontal	2.8
	R rostral middle frontal and R superior frontal	2.3
	R caudate and R superior frontal	2.1
	R superior frontal and R insula	2.1
2	L thalamus and L superior frontal	5.4
	L rostral middle frontal and L superior frontal	3.7
	L superior frontal and L caudate	3.3
	L rostral middle frontal and L caudate	3.2
	L superior frontal and L insula	3.2
	L superior frontal and R superior frontal	3.1
3	R superior parietal and R supramarginal	3.5
	R inferior parietal and R superior parietal	3.2
	R inferior parietal and R middle temporal	3.0
	R thalamus and R superior parietal	2.4
	R lateral occipital and R middle temporal	2.2
4	L lateral occipital and L superior temporal	3.5
	L precuneus and L thalamus	3.3
	L superior parietal and L thalamus	2.7
	L superior parietal and L superior temporal	2.3
	L inferior parietal and L precentral	2.2
5	L superior frontal and R superior frontal	6.4
	L rostral middle frontal and R superior frontal	5.4
	L rostral middle frontal and R rostral middle frontal	4.6
	L superior frontal and R rostral middle frontal	4.3
	L superior parietal and R superior parietal	2.5

List derived from “regionconnect” tool running on the IIT Human Brain Atlas (v.5.0). The last column shows the % probability that a streamline passing through a voxel of the cluster belongs to a certain connection. Connections with lower probabilities not shown.

## Discussion

This study examined the association between late-life depressive symptoms and white matter structural integrity in a large epidemiological sample of older Black participants without dementia. The results showed that, after correcting for age and other relevant covariates, higher level of self-reported late-life depressive symptoms was associated with reduced white matter integrity across numerous pathways, a well-established finding in the general literature. For a finer localization, regionconnect was used to identify the most probable connections running through white matter regions with significant findings. The connections that emerged included commissural pathways between contralateral prefrontal regions (superior and middle frontal/DLPFC) and association pathways connecting DLPFC with insular, striatal and thalamic regions, as well as association pathways connecting the parietal, temporal and occipital lobes and the thalamus. The associations emerged with the trace of the diffusion tensor indicating increased mobility of water molecules and suggesting degeneration of normal barriers to free diffusion as one of these white matter mechanisms. Significant associations between depressive symptoms and trace, but not FA, may have been observed for two reasons. First, depressive symptoms may be associated with a relatively uniform increase in diffusivity in all directions, which can increase the trace without affecting FA. Second, the biological changes in trace that were associated with depressive symptoms may have been more substantial than any biological changes in FA. Thus, for the available statistical power, trace changes may have been sufficiently high to exceed the value of p threshold for significance, while FA changes may have not been high enough to reach significance.

The results suggest that reduced white matter integrity in a DLPFC-striatal-limbic-thalamic circuit forms a prominent segment of the neurobiological substrate underlying depressive symptoms in older Black adults. This circuitry is critical for cognitive (particularly executive) function, motor/psychomotor function, and mood regulation (Leh et al., 2007; Drevets et al., 2008; Wen et al., 2014; Kim and Han, 2021), and reductions in white matter integrity along its fibers and projections is commonly linked to late-life depressive symptoms in the general literature (Wen et al., 2014; Smagula and Aizenstein, 2016; Van Agtmaal et al., 2017; Alexopoulos, 2019; Coloigner et al., 2019; Kim and Han, 2021). Another prominent segment of this neurobiological substrate emerged in regions of intra-parietal connections and in connections between parietal/temporal/occipital regions and thalamic nuclei. This posterior circuitry contributes to a vast array of complex functions (Culham and Kanwisher, 2001), many of which are relevant to depressive symptoms including sensory and affective aspects of pain (Benuzzi et al., 2008), reactivity to environmental cues (Meyer and Bucci, 2016), and self-awareness of action (Blakemore and Frith, 2003), and, along with connections to DLPFC and related regions, this segment comprises a frontoparietal circuit that has been implicated in the cognitive and emotional symptoms of late-life depression (Tadayonnejad and Ajilore, 2014; Alexopoulos, 2019). Although associations of late-life depressive symptoms with reduced white matter integrity did not emerge in this study for direct connections between DLPFC and parietal regions, the results clearly showed the association in connections to these two high-traffic regions (Bullmore and Sporns, 2009; Gong et al., 2009).

The association between late-life depressive symptoms and white-matter diffusion characteristics was robust even when DTI models were adjusted for whole-brain and voxel-wise WMHs, suggesting that altered white-matter diffusion above and beyond visible macrostructural injury is at play in this association. In the current study, the association emerged with the trace of the diffusion tensor which assesses the amount of diffusion of water molecules without regard for directionality. Higher trace may signal lower structural integrity of tissue, possibly due to degeneration of normal barriers to free diffusion. Increased trace has been shown to be related to higher systolic blood pressure in healthy older (White) men with normal-appearing white matter (Maclullich et al., 2009), suggesting that alterations in white matter structural integrity due to vascular risk factors may occur before there is any evidence of other structural injury. Here we showed that subclinical depressive symptoms were associated with altered white-matter diffusion after controlling for white-matter hyperintensities. It is possible that pre-visible alterations in white matter structural integrity in healthy older adults, and in particular healthy older Black adults who are at higher risk for vascular conditions, could make an early contribution to a continuum of white-matter alterations that interrupt connectivity between regions in a highly distributed network that supports affective health, and create a predisposition to late-life depressive symptoms. Longitudinal studies will be necessary to examine this hypothesis.

The association between late-life depressive symptoms and reduced white matter integrity demonstrated in this study is particularly relevant to Black adults who have higher rates of vascular risk factors and vascular disease (Carnethon et al., 2017; Brothers et al., 2019) and higher WMH burden (Brickman et al., 2008; Whitlow et al., 2015; Zahodne et al., 2015) that could potentially diminish connectivity along these circuits and lead to compromise in cognition, motor/psychomotor function, and mood. Indeed, vascular depression is clinically defined by the presence of cognitive impairment, psychomotor retardation, depressive symptoms, and MRI-evidence of vascular disease (Krishnan et al., 1997; Alexopoulos, 2019), and Black adults may thus be predisposed to this diagnosis (Reinlieb et al., 2014), especially in late-life when age and common vascular risk factors such as hypertension and diabetes increase risk of WMHs (Gottesman et al., 2010; Nyquist et al., 2014; Whitlow et al., 2015; Hughes et al., 2018; Waldron-Perrine et al., 2018). However, little remains known about the construct of vascular depression and its correlates in older Black adults due to a stark paucity of research (Bogoian and Dotson, 2022). To validate the construct of vascular depression within Black adults, and to understand its correlates to the extent that race-relevant interventions could potentially be developed, will require more studies that include neuroimaging with behavioral measures and employ large epidemiologic all-Black samples.

It is important to note that the CES-D scale used in this study measures subclinical depressive symptoms that do not reach the threshold of clinically-diagnosed major depressive disorder (MDD). Subclinical depressive symptoms increase the risk of developing MDD (Cuijpers and Smit, 2004; Tuithof et al., 2018) and share many of the same adverse outcomes (Beekman et al., 1997; Ayuso-Mateos et al., 2010), suggesting that they occupy a transitional position between euthymic mood and MDD (Zhang et al., 2020). However, not all persons with subclinical depressive symptoms go on to develop MDD (Tuithof et al., 2018), and it is possible that the coupling of depressive symptoms with altered brain structure may aid in identifying those who are most at risk of developing MDD and its attendant adverse outcomes. Indeed, older Black adults report high levels of subclinical depressive symptoms but have *low* rates of diagnosed MDD. This “race paradox in mental health” (Mezuk et al., 2013; Barnes and Bennett, 2014) may be explained by individual differences in sociodemographic and psychosocial risk and protective factors including socioeconomic status, access to mental health treatment, various sources of stress exposure (discrimination, trauma), and psychological resiliency that influence the probability of MDD diagnosis in Black adults (Tobin, 2021). Individual differences in brain integrity might add further explanatory power; perhaps it is only those older Black adults who have certain stress exposures concomitant with compromised white matter integrity that are at highest risk of transitioning to a diagnosis of MDD. While longitudinal studies are needed to replicate and validate this association, it may hold promise as a race-relevant prognostic feature of movement along the spectrum of late-life depression, potentially aiding in early detection of those individuals who are at risk of MDD, and thus optimizing treatment and reducing racial disparities in affective and brain health.

This study had some limitations. First, the models did not adjust for health-related or sociocultural factors. The purpose of this study was to first determine if there is indeed a link between late-life depressive symptoms and reduced white matter structural integrity within older Black adults. Having accomplished that, future studies will examine potential cognitive, medical, sociocultural, biological, and lifestyle moderators of this important association. Further, the regions identified here can be examined in future studies as potential biological mediators of associations between late-life depressive symptoms and important adverse outcomes. Second, the participants in this study reside in the Chicagoland area and are mostly women with an education beyond high school, and thus the results may not generalize to the US community-dwelling, or international older Black population. Third, the study was cross-sectional. Longitudinal studies showing a link between change in depressive symptoms with change in MRI are required before making any conclusions regarding direction of causality. Finally, even though the average number of depressive symptoms was low in the analytic sample, the frequency was not that different from other published work (Skarupski et al., 2005; Barnes et al., 2009, 2012a,b; Wilson et al., 2010; Turner et al., 2015), and could reflect a healthy volunteer effect in cohort studies. Importantly, despite the low frequency of depressive symptoms, a clear pattern of association with reduced structural integrity was demonstrable within a minoritized population that is underrepresented in research and underserved clinically. Future studies are needed to build upon this finding. These limitations notwithstanding, this study had important strengths. First, neuroimaging studies focusing on variation within Black samples are sorely needed but remain exceedingly rare. Second, the sample, which was well-characterized, was drawn from three large and well-established epidemiologic studies of aging and dementia whose recruitment and data collection procedures are harmonized. Third, by controlling for WMHs on a whole-brain and voxel-wise basis, we were able to measure white matter structural compromise over and above the effects of WMHs. Finally, using regionconnect software, we were able to identify the most probable white matter connections traversing regions where abnormal structural integrity was linked to late-life depressive symptoms in older Black adults.

In conclusion, the results of this study demonstrated that late-life depressive symptoms are associated with a discernable pattern of reduced white matter structural integrity in older Black adults. Future studies are needed to build upon these findings with the goal of deepening understanding of within-Black variability in brain-behavior relationships and ultimately applying that knowledge to reduce racial disparities in affective health and treatment.

## Data availability statement

The datasets presented in this study can be found in online repositories. The names of the repository/repositories and accession number (s) can be found at: www.radc.edu.

## Ethics statement

The studies involving human participants were reviewed and approved by Institutional Review Board of Rush University Medical Center. The patients/participants provided their written informed consent to participate in this study.

## Author contributions

DF, KA, SL, and LB: study concept and design and drafting of the manuscript. LB, DB, and KA: acquisition of data. DF, KA, SL, SZ, and LB: analysis and interpretation of data. DF, KA, SL, SZ, ML, SH, VP, NK, DB, and LB: revision of the manuscript for important intellectual content. SL and SZ: statistical analysis. KA, ML, SH, VP, LB, and DB: obtained funding. All authors contributed to the article and approved the submitted version.

## Funding

This work was supported by National Institute on Aging grants R01AG56405, R01AG22018, P30AG10161, P30AG72975, R01AG17917, R01AG064233, R01AG052200, R01AG055430, R01AG062711, and K01AG064044; National Institute of Neurological Disorders and Stroke UH3NS100599; and by the Illinois Department of Public Health.

## Conflict of interest

The authors declare that the research was conducted in the absence of any commercial or financial relationships that could be construed as a potential conflict of interest.

## Publisher’s note

All claims expressed in this article are solely those of the authors and do not necessarily represent those of their affiliated organizations, or those of the publisher, the editors and the reviewers. Any product that may be evaluated in this article, or claim that may be made by its manufacturer, is not guaranteed or endorsed by the publisher.

## Footnotes

^1^ www.nitrc.org/projects/iit
